# Unrealistic Optimism in the Time of Coronavirus Pandemic: May It Help to Kill, If So—Whom: Disease or the Person?

**DOI:** 10.3390/jcm9051464

**Published:** 2020-05-13

**Authors:** Dariusz Dolinski, Barbara Dolinska, Barbara Zmaczynska-Witek, Maciej Banach, Wojciech Kulesza

**Affiliations:** 1 Faculty of Psychology in Wroclaw, SWPS University of Social Sciences and Humanities, 53-238 Wroclaw, Poland; 2 Faculty of Social Sciences, University of Opole, 45-052 Opole, Poland; bddolinska@swps.edu.pl (B.D.); zmacz@uni.opole.pl (B.Z.-W.); 3 Department of Hypertension, WAM University Hospital, Lodz, Medical University, 93-338 Lodz, Poland; Polish Mothers Memorial Hospital Research Institute (PMMHRI), 93-338 Lodz, Poland; 4 Faculty of Psychology in Warsaw, SWPS University of Social Sciences and Humanities, 03-815 Warsaw, Poland

**Keywords:** unrealistic optimism, unrealistic pessimism, risk perception, healthy illusion, threat and fear

## Abstract

**Objective**: The results of numerous empirical studies have showed the occurrence of so-called unrealistic optimism. Thus, we aimed to investigate whether in the situation of an imminent coronavirus pandemic, people would still perceive themselves as being less exposed to the disease than others. **Methods**: Survey studies were conducted to examine the level of unrealistic optimism. Participants (*n* = 171, 67.3% of women) in a subjective way judged the risk of their coronavirus infection and the likelihood that this would happen to an average student of the same sex from their class. The survey was conducted in three waves: prior to the announcement of the first case of coronavirus (2–3 March), immediately after that announcement (5–6 March), and a few days later (9–10 March). **Results**: We showed that women estimated the chances of being infected as significantly higher (*M* = 4.52, *SD* = 2.079; *t* = 2.387; *p* = 0.018; *Cohen’s d* = 0.393) than men (*M* = 3.71, *SD* = 2.042). The phenomenon of unrealistic optimism was observed especially in men (as compared to other male participants) as it appeared in all three measures (*M* (you) = 3.95 vs. *M* (other male student) = 4.63; *M* = 3.71 vs. *M* = 4.68, and *M* = 4.46 vs. *M* = 5.38 in phase one, two, and three, respectively; *p* ≤ 0.006 for all comparison), but also in women in the last two measures (*M* (you) = 4.55 vs. *M* (other female student) = 4.95, and *M* = 4.99 vs. *M* = 5.38 in phase 2 and 3, respectively; *p* ≤ 0.012 for both comparisons). **Conclusions**: The study revealed a fairly general occurrence of unrealistic optimism, which was mainly observed in men as it appeared in all three measures, but also in women in the last two measures. This result is important for health experts who are responsible for making people comply with regulations concerning social distancing, putting masks on to stop infection, and staying at home. It is possible that unrealistically optimistic people will behave much less in line with the aforementioned recommendations, causing coronavirus to spread widely.

## 1. Introduction

People are frequently engaged in making predictions about various aspects of their own future, including health aspects. This is important for them in terms of formulating goals and making decisions. Pessimists expect rather unfavorable developments, while optimists usually think that events will develop in a manner that is beneficial to them. Numerous psychological studies have demonstrated that optimism is, generally speaking, associated with better social and task-related functioning and increases the chances of achieving successes [1,2]. A wealth of psychological studies also indicates a link between optimism and physical health. For example, optimists report various health problems less frequently than do pessimists [3,4], after coronary bypass surgery they make faster progress during the recovery period and experience less severe anginal pain [5], they also exhibit high blood pressure less frequently than pessimists [6]. Scheier and Carver [7], in a review of studies on the positive relation between optimism and physical health, show that the beneficial impact of optimism on health is the result of two processes. First, optimism makes people more inclined to undertake a range of health promoting behaviors. Second, positive expectations influence a person’s underlying biological responses (including immunological one) to stress and stressors, which ultimately promotes better physical health. 

A specific form of optimism is the way in which people compare themselves with others regarding the probability of experiencing different and realistically possible positive and negative states in the future. The results of numerous empirical studies show the occurrence of so-called *unrealistic optimism*—a bias where “people believe that negative events are less likely to happen to them than to others, and they believe that positive events are more likely to happen to them than to others” [8]. Numerous studies show that unrealistic optimism affects a range of aspects of human life. Of interest to us, it is worth mentioning that this effect has been found in relation to the probability of experiencing various diseases, such as alcoholism or heart attack [9], breast cancer in women, and prostate cancer in men [10]. Taylor and Brown [11] in their seminal article defined unrealistic optimism as part of a pattern of so-called positive illusions that help us cope with potentially threatening experiences.

Although people are usually unrealistically optimistic, this is not always the case. For example, Dolinski, Gromski, and Zawisza [12] undertook a study on Polish students a week after the tragic accident at the Chernobyl nuclear power plant, when a radioactive cloud had just arrived over Poland and was expected to endanger citizens’ health and life. The students were asked to estimate the probabilities that they might experience various negative states (such as heart attack, being robbed, or being in a train crash) and the probabilities that an average student of their gender might encounter such problems. The results clearly indicated the occurrence of unrealistic optimism—the participating students believed that these bad things were less likely to happen to them than to the average student. However, when they were asked about the probability of falling ill with radiation sickness as a result of experiencing a highly excessive dose of radiation, the exact opposite effect was noted. The participants judged that they were more vulnerable than others to the sickness. The researchers termed this effect “unrealistic pessimism”. This study is most likely the only one in history where unrealistic optimism was examined at a time when the participants were experiencing a state of general, unexpected, and new (for them) threat that might affect their health and even life expectancy.

The emergence of the coronavirus pandemic is also a phenomenon that affects everyone (in general) and just like the abovementioned Chernobyl disaster, is new and, at least to some extent, unexpected. Therefore, we wanted to find out whether in the situation of an imminent coronavirus pandemic, participants would perceive themselves as being exposed to the disease to the same degree as the average person from their reference group or whether their beliefs would be biased. In the latter case, the question arises in turn as to whether this bias would be one of unrealistic optimism or rather unrealistic pessimism. Moreover, we aimed to examine the dynamics of beliefs about the likelihood of experiencing coronavirus infection. It could be assumed that such beliefs would be influenced by media coverage of the spread of the infection in the country where the study was conducted and information about the situation in other countries.

## 2. Materials and Methods

### 2.1. Study Design

While planning our research, we came to the conclusion that from this perspective, the official announcement of the first case of the disease in Poland in the media would be an exceptionally important event. Simply put, after the announcement, the serious threat already exists; before, it is possible to establish a “starting point”. Making it a priority to start our research before this occurred, we decided to minimize the number of variables included in the research design, limiting ourselves to the sole issue of unrealistic optimism/pessimism. As a result, the time needed for completing the survey was less than a minute, which, in turn, made it possible for us to obtain the consent of lecturers to conduct the research during classes. Thus, we were able to initiate our program immediately after posing the above-mentioned research questions.

The first phase of our study took place on 2–3 March 2020. Of course, prior to this date, Polish citizens were already expecting that the coronavirus would be found in their country in the coming days, mainly because a great number of cases had already been reported in Germany, which borders Poland. However, as of 3 March, there was not a single confirmed case in Poland. That same day, in the afternoon (after we had finished compiling the results of the first phase of the study), Poland’s Minister of Health officially announced that there were around 100 patients hospitalized in Polish hospitals at that time with suspected coronavirus (official test results were yet to be announced) and 300 more people were being quarantined. With this information, people went *en masse* to shops, purchasing huge reserves of mainly personal hygiene products, rice, groats, and pasta. Many shops also ran out of meat. The next morning (4 March) a special press conference was called at which the Minister of Health announced that the first case (patient “0”) of coronavirus in Poland was discovered in a man who had come from Germany a few days earlier. 

This led us to decide to immediately carry out (i.e., on 5 and 6 March) the second phase of the study. In the next two days (i.e., 7–8 March), several new cases were reported in different places in Poland. This also coincided with reports of a sharp increase in morbidity and deaths in various European countries. It was also announced that coronavirus infections had already been reported in 104 countries worldwide. The Minister of Health clearly stated that the number of infected people was expected to increase rapidly in the coming days. On 9 and 10 March, we carried out the final third phase of our study. The following day, on 11 March, the University of Opole (as well as other Polish universities) was closed down and teaching activities halted.

### 2.2. Procedure

To make the sample as wide as possible, students of four faculties (psychology [year of study 1 & 2], law [year 3], internal security [year 2], IT [year 1 & 2]) at the University of Opole were invited to take part in the study (they were informed that it was voluntary, anonymous, and would last about one minute). Making contact with students was made possible thanks to the courtesy of lecturers who had previously expressed their consent. As was already described above, the survey was conducted in three waves: 2–3 March (i.e., before the announcement of the first coronavirus case in Poland), 5–6 March (i.e., immediately after the announcement of the first case), and 9–10 March 2020 (after multiple cases had already been confirmed and the Minister of Health definitively stated that a sharp increase in the number of infected people should be expected). Each time the participants were given a piece of paper on which they were asked to write down their gender as well as a code consisting of the first letter of their father’s forename, the first letter of their mother’s forename, and the number corresponding to the month of their birth. This procedure, on the one hand, ensured the anonymity of the study participants and on the other, allowed us to attribute the next three completed surveys (in individual phases of the study) to a given person.

The participants were asked to respond to the following two questions:

1. How likely is it that you will become infected with coronavirus?
2. How likely is it that the average student of your sex in your class will become infected with coronavirus?

In both cases, the students estimated the likelihood on an 11-point scale, from 1—“*entirely impossible*” to 11—“*definitely*”.

This research was approved by the Ethical Review Committee of the Faculty of Psychology in Wroclaw, SWPS University.

### 2.3. Statistical Analysis 

Student’s *t* test for dependent comparisons was used while analysing the obtained results with mean (M) values ± standard deviation (*SD*). For the dependent samples *t*-test, *Cohen’s d* was determined by dividing the mean of differences by the SD of the differences.

## 3. Results

A total of 432 participants were engaged, but since lectures (as opposed to other instructional formats, such as exercises or seminars) are not obligatory at the University of Opole, the same people did not always appear at the individual classes. Some students were also late for a lecture or were in a great hurry after its completion, thus they did not participate in all three stages. The analyses presented below were therefore only carried out on the group of those students who responded to both questions on three occasions. Thus, finally, 171 participants (115 women [67.3%] and 56 men) participated in our study. Preliminary analyses showed that the field of study being pursued by the respondents did not differentiate the answers (*p* > 0.05). There were also no differences between the results obtained during the two days comprising a given phase of the study.

However, some gender differences emerged in estimations of the probability of falling ill: during the second measure, women estimated their own probability higher: *M* = 4.52 (standard deviation [*SD*] = 2.079) than did men: *M* = 3.71 (*SD* = 2.042); *t* = 2.387; *p* = 0.018; *Cohen’s d* = 0.393. Due to this difference and the slightly distinct dynamics of unrealistic optimism in both genders, further analyses concerning the estimates made by participants are presented below separately for women and men.

### 3.1. Dynamics of Unrealistic Optimism in Women

In the first phase of the study, estimates concerning the likelihood of oneself falling ill (*M* = 4.29; *SD* = 2.085) and of illness occurring in the average student of the same sex (*M* = 4.45, *SD* = 1.827) were similar enough (*t* = 1.045) that we cannot say an unrealistic optimism effect is present. It did, however, appear in the second phase of the study: *t* = 2.554; *p* = 0.012; *Cohen’s d* = 0.238 for *M* (female participant) = 4.55 (*SD* = 2.062) vs. *M* (other female student) = 4.95 (*SD* = 2.038) and became more pronounced in the third phase: *t* = 2.793; *p* = 0.006; *Cohen’s d* = 0.260 for *M* (female participant) = 4.99 (*SD* = 2.466) vs. *M* (other female student) = 5.38 (*SD* = 2.434).

**Figure 1:**
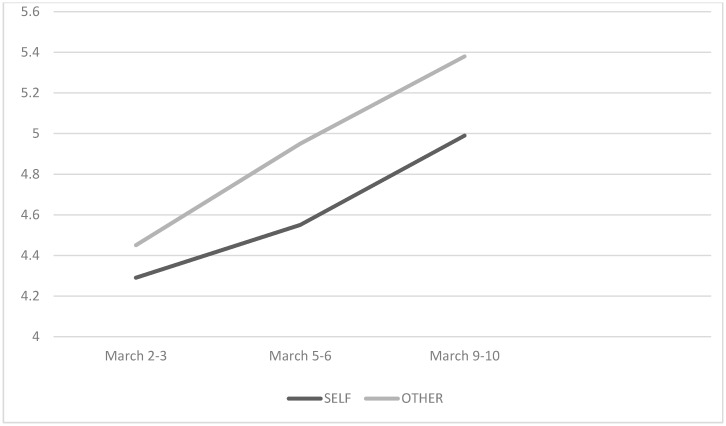
Women’s estimates of the likelihood of contracting coronavirus and the likelihood that an average student of the same sex will be infected at three different time intervals.

Regarding the dynamics of perception of the probability of one’s own illness, we note a consistent drop in optimism. There is a statistically significant deterioration in the prediction between the first (*M* = 4.29; *SD* = 2.085) and second measure (*M* = 4.55, *SD* = 2.062; *t* = 2.080; *p* = 0.040; *Cohen’s d* = 0.193), as well as between the second (*M* = 4.55, *SD* = 2.062) and the third (*M* = 4.99; *SD* = 2.466; *t* = 2.873; *p* = 0.005; *Cohen’s d* = 0.290). The situation is similar as regards the perception of the likelihood that another average female student will get ill. The difference between the first (*M* = 4.45, *SD* = 1.827) and second measure is (*M* = 4.95, *SD* = 2.038; *t* = 3.752; *p* = 0.001; *Cohen’s d* = 0.353), while between the second (*M* = 4.95, *SD* = 2.038) and third (*M* = 5.38, *SD* = 2.434; *t* = 2.863; *p* = 0.005; *Cohen’s d* = 0.264) (Figure 1).

### 3.2. Dynamics of Unrealistic Optmism in Men

In men, unrealistic optimism manifested at the beginning of the study and was observed consistently during the next two measures. Estimates of the likelihood of own illness and the average male student’s illness were different in all three phases. In the first phase: *t* = 2.853; *p* = 0.006; *Cohen’s d* = 0.382 for *M* (male participant) = 3.95 (*SD* = 2.058) vs. *M* (other male student) = 4.63 (*SD* = 2.245). In the second phase: *t* = 2.869; *p* = 0.006; *Cohen’s d* = 0.386 for *M* (male participant) = 3.71 (*SD* = 2.042) vs. *M* (other male student) = 4.68 (*SD* = 2.367). In the third phase: *t* = 3.642; *p* = 0.001; *Cohen’s d* = 0.491 for *M* (male participant) = 4.46 (*SD* = 2.374) vs. *M* (other male student) = 5.38 (*SD* = 2.707).

As regards the dynamics of perception of the participant himself falling ill, this decreased slightly (without statistical significance: *t* = 1.032) in the second measure, but increased in the third measure. The increase in pessimism concerning one’s own health reached a statistically significant level; *t* = 2.579; *p* = 0.013; *Cohen’s d* = 0.345. The difference between the first and third estimate did not differ significantly (*t* = 1.498).

**Figure 2:**
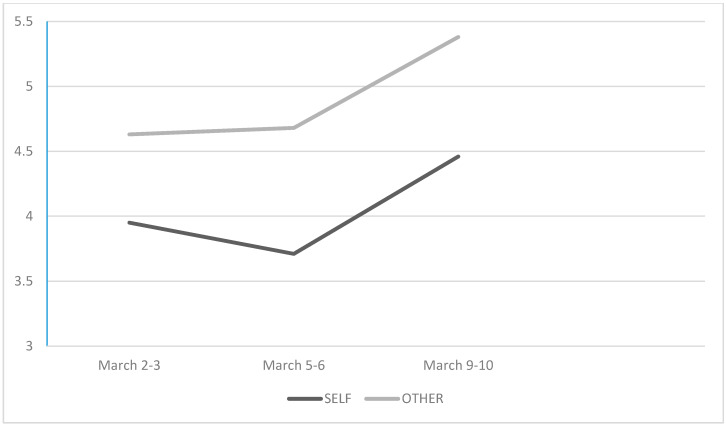
Men’s estimates of the likelihood of contracting coronavirus and the likelihood that an average student of the same sex will be infected at three different time intervals.

At the same time, it turned out that male students estimating the probability of the average student of the same sex falling ill indicated this possibility as very similar in the first and second phase (*t* < 1), with their estimates only increasing during the third measure. The differences turn out to be statistically significant when we compare the estimates in the 1^st^ and 2^nd^ (*M* = 4.68, *SD* = 2.367) measure with the estimates in the third measure (*M* = 5.38; *SD* = 2.707; *t* = 2.035; *p* = 0.047; *Cohen’s d* = 0.272, and *t* = 2.668; *p* = 0.010; *Cohen’s d* = 0.358, respectively) (Figure 2).

## 4. Discussion

The study revealed a fairly general occurrence of unrealistic optimism (especially in men as it appeared in all three measures, but also in women in the last two measures). This result is absolutely critical for health experts and officials who are responsible for making people comply with regulations concerning social distancing, putting masks on to stop infection (themselves/others), and staying at home. It is possible that the sample will probably behave much less in line with the aforementioned recommendations, causing coronavirus to spread widely.

The primary question that arises in the context of this regularity is the difference from the pattern obtained in two previous studies conducted in a similar context. We recall that Dolinski, Gromski, and Zawisza [12] studying people who were aware that there was a radioactive and deadly cloud hanging over their heads, who were asked about the probability of falling ill due to its presence, recorded a clear effect of unrealistic pessimism. Burger and Palmer [13], who asked people about the likelihood that they vs. someone else would be victims of an earthquake a week just after such an event, demonstrated the absence of bias (because unrealistic optimism is usually noted, researchers write about the decline of this optimism in conditions where negative memories remain quite vivid). However, in the research presented here, we have obtained completely different results: unrealistic optimism not only appeared in the men surveyed in the first phase of our study, but persisted in the subsequent two phases. This was also true in the women, especially in the last two measures. In particular, the size effects, measured as *Cohen’s d*, varied from 0.238 up to 0.491, meaning that the effect varies from a “small effect size” to a “moderate effect size”.

The question thus arises: what is the difference between the situation we are investigating and a nuclear power plant failure or an earthquake? The studies on unrealistic optimism have showed that an important factor influencing the magnitude of this effect is the belief in the controllability of a given event. The effect of unrealistic optimism is clearly stronger in relation to events that people think they have influence over [14,15,16] Both of the previously examined situations seem to be associated with a sense of lack of controllability. The communist authorities in Poland first concealed the fact of the explosion of the nuclear power plant in Chernobyl and then minimized the negative consequences of this event and the ensuing threat. People did not know whether there were any means of reducing the real probability of getting ill. As far as an earthquake is concerned, it is not a very predictable phenomenon (especially in the somewhat longer term) and there are few possibilities to reduce individual danger by individual actions.

The case we investigated during the coronavirus pandemic is different. First of all, the element of surprise is smaller. Residents of Poland first heard a lot about the coronavirus spreading in China, then they gradually received information about its appearance in various European countries, and only after a significant period of time, they learnt that it had also appeared in Poland. Additionally, people were informed that frequent handwashing and avoiding larger groups of people (or even better, if possible, staying at home) could clearly decrease the probability of getting ill. Finally, from the very beginning, they received the information that mainly older people are at risk and 80% of cases might be asymptomatic or with mild symptoms. The conviction of the (at least partial) controllability of this event may therefore have encouraged the development of a positive bias, consisting of the conviction that one is less at risk than the average student of one’s own gender.

Another question related to the pattern of results we obtained is perhaps even more important: whether the unrealistic optimism revealed in our research is a result to be celebrated or whether it is a prediction of people’s poor performance. Studies on the relationship between unrealistic optimism and well-being, good mood, or life satisfaction indicate positive consequences of experiencing this illusion [11,17]. However, research on the relationship between unrealistic optimism and physical health is less conclusive. On the one hand, there are data indicating its positive role—unrealistic optimism was conducive to the emergence of health promoting behavior [18], on the other hand, there are data indicating its destructive role. Studies have shown, for example, that unrealistic optimism made patients reluctant to undergo medical treatment [19] that we would rather be dealing with the first of these regularities (in fact, there are observations suggesting that unrealistic optimism has been observed in young people 20 and 30+) than what was the reason for the failure of complying with the sanitary orders and fast virus transmission [19,20,21]. We note that both women (consistently) and men from the third measure give higher estimates of the probability of their own infection with coronavirus. So, we can say that in our participants, we see the phenomenon of illusion rather than delusion. If we treat the higher estimated probability of falling ill compared to a few treatment [19] or disregarding the possibility of alcoholism, they acquired addiction [20]. Looking at our results, showing the dynamics of unrealistic optimism in our participants, one can (optimistically!) risk the statement days earlier as an indicator of an increase in awareness of the existing danger, then the simultaneous belief that others are even more exposed than oneself may have positive consequences—this may be associated with a lack of excessive anxiety, leading to passivity [22,23] and motivate engagement in health promoting activities [18]. Of course, we must stress that this is only supposition on our part, unsupported by empirical evidence.

### 4.1. Limitations and Further Questions

Although the dominant pattern of the results in our study is unrealistic optimism, which persists in spite of further alarming or even horrifying information, some gender differences are noted. Women, unlike men, did not reveal unrealistic optimism in the first measure, while men, unlike women, did not react with an increase in pessimism about the chances of getting ill between the first and second measure. While the second of these results can be explained by the fact that information about the first patient infected with coronavirus in Poland scared men less than women, in relation to the first question (i.e., the absence of the effect of unrealistic optimism in women in the first phase), we simply must admit that we cannot identify any sensible explanation.

An undoubted weakness of our research is that it stops with the third measure—simply (since the universities in Poland were closed), we could not proceed with the anonymous survey with the same sample group of students/we have lost contact with this group. It cannot be ruled out that following information about coronavirus fatalities in Poland or the faster and wider spread of the disease than originally assumed, the participants’ belief that they are less vulnerable than other people would change. Unfortunately, the cancellation of classes rendered it impossible to continue our research. Usually, authors in such situations write that they have plans to continue their research and to empirically resolve the issue. We take the liberty of writing that we hope we will not have another opportunity to do so.

### 4.2. Further Research Directions and Recommendations

First and foremost, future studies conducted under circumstances of the COVID-19 pandemic should address the relationship between unrealistic optimism and preventive behavior. There is a deep need to know if unrealistic optimism is directly related to compliance with medical/governmental recommendations—wearing masks and gloves, maintaining physical distance, frequent washing of hands, and other restrictions—or not. With the present data in hand, we do not know if unrealistic optimism affects behavior and in which direction (fulfilling/rejecting recommendations). Secondly, more studies should be run to check to what extent these results would be replicated in other countries. It can be assumed, for example, that especially in countries with a collectivist culture, in which egotism is less evident [24], unrealistic optimism will not occur. Thirdly, it is crucial to undertake research on ways to reduce unrealistic optimism, i.e., fight against this possibly deadly psychological mechanism. Such attempts are already described in the literature [9] (see: Wei, but it is necessary to check whether these methods will also be effective in a specific situation of coronavirus pandemic. Finally, other groups (not, as in this case: students) should be tested to check the generalizability of the effect. For example, the question arises: do medical personnel fall into unrealistic optimism to the same extent, or not?

## References

[1] Matlin M., Stang D. (1978). The Polyanna Principle.

[2] Seligman M. (2002). Authentic Happiness: Using the New Positive Psychology to Realize Your Potential for Lasting Fulfillment.

[3] Scheier M., Carver C. (1985). Optimism, coping and health: Assessment and implications of generalized outcome expectancies. Health Psychol..

[4] Robbins A., Spence J., Clark H. (1991). Psychological determinants of health and performance: The tangled web of desirable and undesirable characteristics. J. Personal. Soc. Psychol..

[5] Fitzgerald T., Tennen H., Affleck G., Pransky G. (1993). The relative importance of dispositional optimism and control appraisal in quality of life after coronary artery bypass surgery. J. Behav. Med..

[6] Raikkonen K., Matthews K., Flory J., Owens J. (1999). Effects of optimism, pessimism, and trait anxiety on ambulatory blood pressure and mood during everyday life. J. Personal. Soc. Psychol..

[7] Scheier M., Carver C. (2008). Dispositional optimism and physical health: A long look back, a quick look forward. Am. Psychol..

[8] Weinstein N. (1980). Unrealistic optimism about future life events. J. Personal. Soc. Psychol..

[9] Weinstein N.D. (1983). Reducing unrealistic optimism about illness susceptibility. Health Psychol..

[10] Clarke V., Lovegrove H., Williams A., Machperson M. (2000). Unrealistic optimism and the health belief model. J. Behav. Med..

[11] Taylor S., Brown J. (1988). Illusion and well-being: A social psychological perspective on mental health. Psychol. Bull..

[12] Dolinski D., Gromski W., Zawisza E. (1987). Unrealistic pessimism. J. Soc. Psychol..

[13] Burger J., Palmer M. (1992). Changes in and generalization of unrealistic optimism following experiences with stressful events: Reactions to the 1989 California earthquake. Personal. Soc. Psychol. Bull..

[14] Zakay D. (1984). The influence of perceived event’s controllability on its subjective occurrence probability. Psychol. Rec..

[15] Klein C., Helweg-Larsen M. (2002). Perceived control and the optimistic bias: A meta-analytic review. Psychol. Health.

[16] Chambers J., Windschitl P. (2004). Biases in social comparative judgments: The role of nonmotivated factors in above-average and comparative-optimism effects. Psychol. Bull..

[17] Jones S., Gell N., Roth J., Scholes D., LaCroix A. (2015). The relationship of perceived risk and biases in perceived risk to fracture prevention behavior in older women. Ann. Behav. Med..

[18] Davidson K., Prkachin K. (1997). Optimism and unrealistic optimism have an interacting impact on health-promoting behavior and knowledge changes. Personal. Soc. Psychol. Bull..

[19] Treloar C., Hopwood M. (2008). Look, I’m fit, I’m positive and I’ll be all right, thank you very much: Coping with hepatitis C treatment and unrealistic optimism. Psychol. Health Med..

[20] Dillard A., Midboe A., Klein W. (2009). The dark side of optimism: Unrealistic optimism about problem with alcohol predicts subsequent negative event. Personal. Soc. Psychol. Bull..

[21] Rothe C., Schunk M., Sothmann P., Bretzel G., Froeschl G., Wallrauch C., Zimmer T., Thie V., Janke C., Guggemos W. (2020). Transmission of 2019-nCoV Infection from an asymptomatic contact in Germany. New Engl. J. Med..

[22] Dewberry C., Richardson S. (1990). Effect of anxiety on optimism. J. Soc. Psychol..

[23] Dewberry C., Ing M., James S., Nixon M., Richardson S. (1990). Anxiety and unrealistic optimism. J. Soc. Psychol..

[24] Nisbett R.E. (2003). The Geography of Thought.

